# Understanding the evolutionary trend of intrinsically structural disorders in cancer relevant proteins as probed by Shannon entropy scoring and structure network analysis

**DOI:** 10.1186/s12859-018-2552-0

**Published:** 2019-02-04

**Authors:** Sagnik Sen, Ashmita Dey, Sourav Chowdhury, Ujjwal Maulik, Krishnananda Chattopadhyay

**Affiliations:** 1 Department of Computer Science and Engineering, Jadavpur University, Kolkata, 700032 India; 2 Chemistry and Chemical Biology, Harvard University, 12 Oxford Street, Cambridge, Massachusetts, 02138 USA; 3 CSIR-Indian Institute of Chemical Biology, Raja S.C. Mullick Road, Kolkata, 700032 India

**Keywords:** Shannon entropy scoring, Multiple sequence alignment, Structure network model, Hydrophobicity index

## Abstract

**Background:**

Malignant diseases have become a threat for health care system. A panoply of biological processes is involved as the cause of these diseases. In order to unveil the mechanistic details of these diseased states, we analyzed protein families relevant to these diseases.

**Results:**

Our present study pivots around four apparently unrelated cancer types among which two are commonly occurring viz. Prostate Cancer, Breast Cancer and two relatively less frequent viz. Acute Lymphoblastic Leukemia and Lymphoma. Eight protein families were found to have implications for these cancer types. Our results strikingly reveal that some of the proteins with implications in the cancerous cellular states were showing the structural organization disparate from the signature of the family it constitutes. The sequences were further mapped onto respective structures and compared with the entropic profile. The structures reveal that entropic scores were able to reveal the inherent structural bias of these proteins with quantitative precision, otherwise unseen from other analysis. Subsequently, the betweenness centrality scoring of each residue from the structure network models was resorted to explore the changes in dependencies on residue owing to structural disorder.

**Conclusion:**

These observations help to obtain the mechanistic changes resulting from the structural orchestration of protein structures. Finally, the hydropathy indexes were obtained to validate the sequence space observations using Shannon entropy and in-turn establishing the compatibility.

**Electronic supplementary material:**

The online version of this article (10.1186/s12859-018-2552-0) contains supplementary material, which is available to authorized users.

## Background

Biological processes are the results of highly orchestrated interactions among the biological macro-molecules. of A majority of these processes occurring within the confines of cell cytosol pivot around the coordinated functions of protein molecules. Encoded by the one-dimensional code script of DNA, the biological relevance of protein molecules reside on their three-dimensional structures. These three-dimensional protein structures arise after a series of sub-structural interactions primarily dictated by sequence information of the protein molecules. In many cases, proteins do not have a stable three-dimensional structures. These proteins are broadly known as Intrinsically Disordered Proteins (IDPs) [1, 2]. IDPs become interesting for the researchers, due to their diverse biological roles and apparent revocation of traditional structure-function paradigm. Regardless of the lack of three-dimensional structure, different biophysical techniques evidenced that IDPs actively participated in various biological processes like control of cell cycle, transcriptional activation, signaling, and they frequently interacted with or functioned as central hubs in protein interaction networks [3].

Proteins are being folded to perform specific functions. Sometimes acquired orderded globular structure may be accompanied by interaction with other proteins. The folding mechanism can be driven by different changes in protein environment. Since proteins are actively involved in different biological processes, a loss of protein structure and disruption in associated interactions can lead to a series of metabolic disruptions in turn inducing a pathological state [4]. A wide range of diseases are caused due to the misfolding of proteins [5]. Misfolding or misfolding function can develop from point mutation or an exposure to internal or external toxins, impaired post-translational modifications (PTMs) [6], an increased probability of degradation, impaired trafficking, oxidative damage or lost binding partners. These factors can act independently or in associations with one another.

Misfolding may cause numerous neurodegenerative and malignant diseases. Reports suggest [7–9], IDPs have an evolutionary significance and correlation with complexity. More elaborately, connection or changes in proteins from most primitive species to modern species can be analyzed depending on the transition from ordered to disordered state or vice versa. The variation in protein residues in protein sequences is responsible for the structural transition which are directly associated with sequence based complexity of the proteins.

Multiple Sequence Alignment (MSA) of a protein family can provide a consensus sequence of that family which might be considered as family sequence representative with the most evolutionarily conserved set of amino acids. As the consensus sequences consist of evolutionarily conserved amino acid residues, so the consensus sequence of a protein family can represent the structural trait for almost all individual members of that protein family. Hence, the complexity score of a consensus in terms of disorder and order can summarize the structural trend of most of the individual proteins from a protein family.

In this article, four diverse cancer types are considered, among which two are well known and frequent malignant diseases viz., Prostate Cancer [10–12], Breast Cancer [13–15], and two relatively less abundant forms viz. Acute Lymphoblastic Leukemia [16, 17] and Lymphoma [17, 18] respectively, along with the proteins responsible for these diseases. Not only the human protein forms are considered, rather the whole family protein sequences are collected in order to compute the MSA and its corresponding consensus sequences. In order to analyze the evolutionary changes, all the sequences of a protein family are studied in details. The Shannon entropy is calculated for the consensus sequence of responsible proteins and also for each sequence from those protein families. Depending on the entropic scores, the proteins were classified as order or disorder in nature. In order to understand the Shannon entropic impact, the sequences are mapped in their respective structure. The hydropathic index is calculated for each member of the protein family in order to compare the sequence complexity in terms of entropic scores. Hence the main motivation of this study is to find the general traits of a protein family in terms of structured and unstructuredness applying complexity scoring.

## Methods

**Fig. 1:**
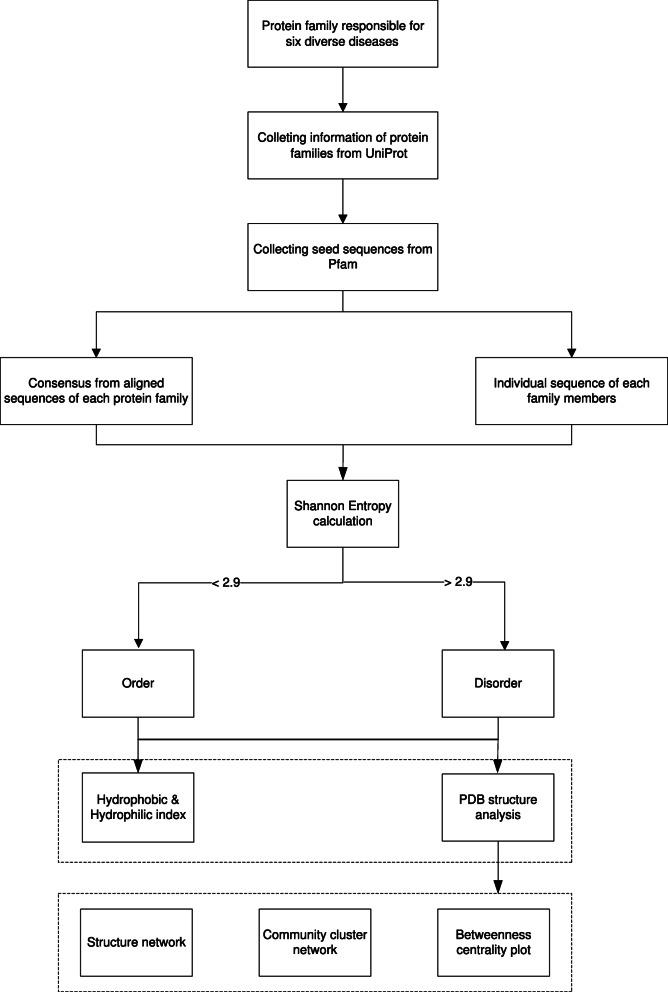
A flowchart to summarize the proposed frame of the work

In this section, we have discussed the proposed framework. Two different databases were used viz., (1) UniProt [19] and (2) Pfam [20]. Initially, eight proteins which were responsible for selected four diverse cancer types, were selected for this study based on frequent occurrence. Later, the sequences of those protein families were considered for further research. In Fig. 1, the flow of the proposed framework is given. The proposed method is discussed below:

### Database information

#### UniProt

UniProt [19] is a cumulative set of sequences and annotated information of these proteins. This database provides around 60 million protein sequences. Since 2014, the database contains around 5631 proteomes. Along with that, the protein family and domain information are described at Uniprot.

#### Pfam

Pfam [20] is another database, consisting of protein family information, multiple sequence alignment and profile hidden Markov models. More than 3000 protein family information is given.

Four cancer types were selected and their commonly responsible protein families in human, depending on the literature survey such as Heat shock protein beta-1 [21, 22], BAG family molecular [23, 24], Breast cancer type 2 [25], Endophilin-B1 [26, 27], Apoptosis regulator Bcl-2 [28, 29], Calpain-type cysteine [30, 31], Cellular tumor antigen p53 [32, 33] and RNA-binding protein 38 [34, 35] were identified from UniProt database. Heat shock protein beta-1 played a role as a molecular chaperone probably maintaining denatured proteins in a folding-competent state. Similarly, BAG family molecular act as a nucleotide-exchange factor, the breast cancer type 2 susceptibility protein (BRCA2) is a breast tumour suppressor involved in double-strand break repair and/or homologous recombination Endophilin-B1 has been observed to regulate the membrane dynamics of various intracellular compartments, Apoptosis regulator Bcl-2 regulates cell death by controlling the mitochondrial membrane permeability, Calpain-type cysteine involved in epiderm development, Cellular tumor antigen p53 acts as a tumor suppressor in many tumor types; induces growth arrest or apoptosis depending on the physiological circumstances and cell type and RNA-binding protein 38 specifically bind the 3’-UTR of CDKN1A transcripts, leading to maintain the stability of CDKN1A transcripts, thereby acting as a mediator of the p53/TP53 family to regulate CDKN1A. Using the Pfam database the aligned sequences of a particular protein family was identified. It was observed that MSA played an important role in comparative functional and structural analysis of biological sequences. In this regard, seed alignment for FASTA format were selected, which included tree ordering sequences and lower case letters were considered with dashes as a gap characteristics. Furthermore, this also provided a biological insight regarding the relationship between the structural and functional behavior of proteins [36]. Therefore, to analyze the type of protein orchestration (i.e., order and disorder) of protein families aligned sequences were considered. Subsequently, a consensus sequence [37] was constructed from the aligned results using Consensus Maker tool. The MSA of each family were provided to this online tool. This tool computed a consensus using customary parameters. These sequences represented as a logo or signature of that protein family, shown in Additional file 1. In this regard, based on the frequency of amino acid i.e the highest frequency of amino acid was considered as an entry of building consensus in that particular position. A consensus sequence is a set of amino acids which are evolutionarily conserved in protein family [38]. For the analyses of the protein sequences present in a particular family, it was necessary to understand the structural orchestration of sequences i.e. propensity towards order and disorder. In this regard, the Shannon entropy (SE) score was calculated for each consensus sequence. As it was evidenced that entropy posses an idea of disorder. Entropy was directly proportional to the rate of disorder i.e. if the disorder increases it signifies higher entropy. Shannon entropy was defined as follows:

1 $$ SE(i)=-\sum\limits_{i=1}^{N}P_{i} {log}_{2} P_{i}  $$

where *P*_*i*_ was the probability of given amino acids and N was the number of letters in a sequence. The summation run over the 20 residues that normally were present in a protein sequence. The probability *P*_*i*_ represent the composition of the consensus sequence. So the entropy range lied between 0 and the *log*_2_(20)=4.32. If the Shannon entropy score of consensus sequences was less than 2.9 then it signify that, particular protein family was ordered [39], on the other hand, if the Shannon entropy was very high then that protein family was disorder and have an important impact on the cause of diseases. The Shannon entropy of each sequence of each family was also calculated in order to validate the results, reported in Additional files 2-9. Moreover, one sample t-test was performed on each protein of a particular family, in order to understand the sample mean which was statistically different from a known or hypothesized population mean. Statistical significance is determined by looking at the *p*-value. The *p*-value gives the probability of observing the test results under the null hypothesis. The lower the *p*-value, the lower the probability of obtaining a result like the one that was observed if the null hypothesis was true. The One Sample t Test is a parametric test. It is defined as:

2 $$ t=\frac{\bar{a}-\mu}{\sigma}  $$

Here, $\bar {a}$ is the sample mean of entropy scores, *σ* is the standard deviation of list entropy scores of a family. *μ* is the specified population mean of list of entropy scores of a family. where,

3 $$ \sigma=\sqrt{\frac{S^{2}}{n}}  $$

*S*^2^ is the sample variance, *n* is the sample size which is total number of proteins from a family. Furthermore, to understand this observation we tried to find the structure of these sequences [40]. These models were further analyzed for structure network analysis. However, complex systems have been analyzed with a help of network models, the interaction between the components of the machines were described through nodes and edges. Generally, secondary structure and folding arrangement mechanism were utilized to understand the protein structures. Another promising method for analysis of the protein structure was through the network [41]. In this network model the amino acid residues represented as nodes and edges which represent the interaction among them, the interaction was established based on the interaction energy or spatial distance. Interactions usually have a weight, which characterized their strength. Depending on this strength the edges were drawn between the two amino acid nodes. The equation was described below.

4 $$ F_{ij}=\left[\frac{x_{ij}}{\sqrt{\left(X_{i}*X_{j}\right)}}\right]*100 \geq F_{c}  $$

*F*_*c*_ was the threshold of interaction strength, the default value is 4%. Here, *x*_*ij*_ was the number of side chain atom pairs of residues *i* and *j*. *X*_*i*_ and *X*_*j*_ were the normalization factor for residues types *i* and *j* [42, 43].

In this paper, depending on the normal mode analysis (NMA) a correlation matrix was obtained in order to perform a cross-correlation matrix. Then by means of correlation network analysis, we generated structure networks [41] of different protein depending on their tertiary structure. The weight of the connection of nodes represented the value of cross-correlation respectively. By means of correlation network analysis, a full residue network was generated and it was split into a highly correlated coarse-grained community cluster network by using Girvan-Newman [44] clustering method where the highly interacting residues were clumped together in the clusters. Here some lower value elements in the raw correlation matrix from NMA were excluded because of being lower than the cutoff value 0.3.

The role of a particular node as a connector between other nodes viz., the importance of a residue to a network in its functioning as a bridging point can be manifested by measuring the number of shortest paths passing through that particular node. Betweenness centrality characterizes the regions of a protein that show differences in coupled motions between networks. Residues having significant contribution to intrinsic dynamics of the protein show high centrality value. Also depending on the centrality scoring, Euclidean distances among the protein mutant types were calculated and the subsequent hierarchical cluster was generated. The betweeness centrality was performed to find the bottlenecks in communication networks and community detection whereas the NMA was performed to generate structure networks of different protein depending on their tertiary structure.

## Results

Reports suggested that selected eight proteins (Endophilin-B1 [26], Breast cancer type 2 susceptibility protein [25], Heat shock protein beta-1 [21], BAG family molecular chaperone regulator 1 [23], Apoptosis regulator Bcl-2 [28], Calpain-type cysteine protease DEK1 [30], Cellular tumor antigen p53 and RNA-binding protein 38 [34]) were associated with four malignant diseases.These proteins were found to have intimate connection with metabolic cascades and interaction networks leading to cancer states.We referred to protein families of these selected proteins so as to understand the generic structural propensity of the protein families which these proteins constitute. To understand the generic structural trend of these protein families, we had performed the Shannon entropy of the consensus sequences. Depending on the entropic score of the consensus sequences, the protein families were being classified as order or disorder. In Table 1, the entropy score of the consensus sequences of each protein families was reported along with the score of t-test [45]. In most of the cases, the protein sequences from a disorder or order class were expected to be disordered or ordered. However, few sequences were reported to be disordered being a part of an ordered protein family in terms of entropic scoring of the consensus protein sequence and vice versa. It is to be noted that, Endophilin-B1 is a responsible protein for breast cancer disease and the entropic score is 2.87. We reported this protein as disorder even though the entropic score is not high but the structural, as well as functional domain, provides an evidence to this. From Protein Data Bank (PDB), we found that the Solution structure of the SH3 domain of Endophilin B1 has higher loops and turns than the number of Beta-coils (PDB Id:1X43). Moreover, the literature studies [26, 27] show the nature of the dynamic functionality of this protein. These evidences supported the disorderedness of this protein as well as our finding.

**Table 1:** The Shannon entropy and t-test value of four selected diverse cancer types

Disease	Protein	Shannon	Significant	*p*-value
	family	entropy	score	
Prostate cancer	Heat shock protein beta-1	3.36	True	7.3e-36
	BAG family molecular	3.39	True	3.4e-40
Breast cancer	Breast cancer type 2	4.01	True	1.0e-30
	Endophilin-B1	2.87	True	5.8e-38
Acute lymphoblastic leukemia	Apoptosis regulator Bcl-2	3.34	True	1.9e-27
	Calpain-type cysteine	2.79	True	3.8e-25
Lymphoma	Cellular tumor antigen p53	4.01	True	1.0e-07
	RNA-binding protein 38	3.38	True	3.8e-96

**Fig. 2:**
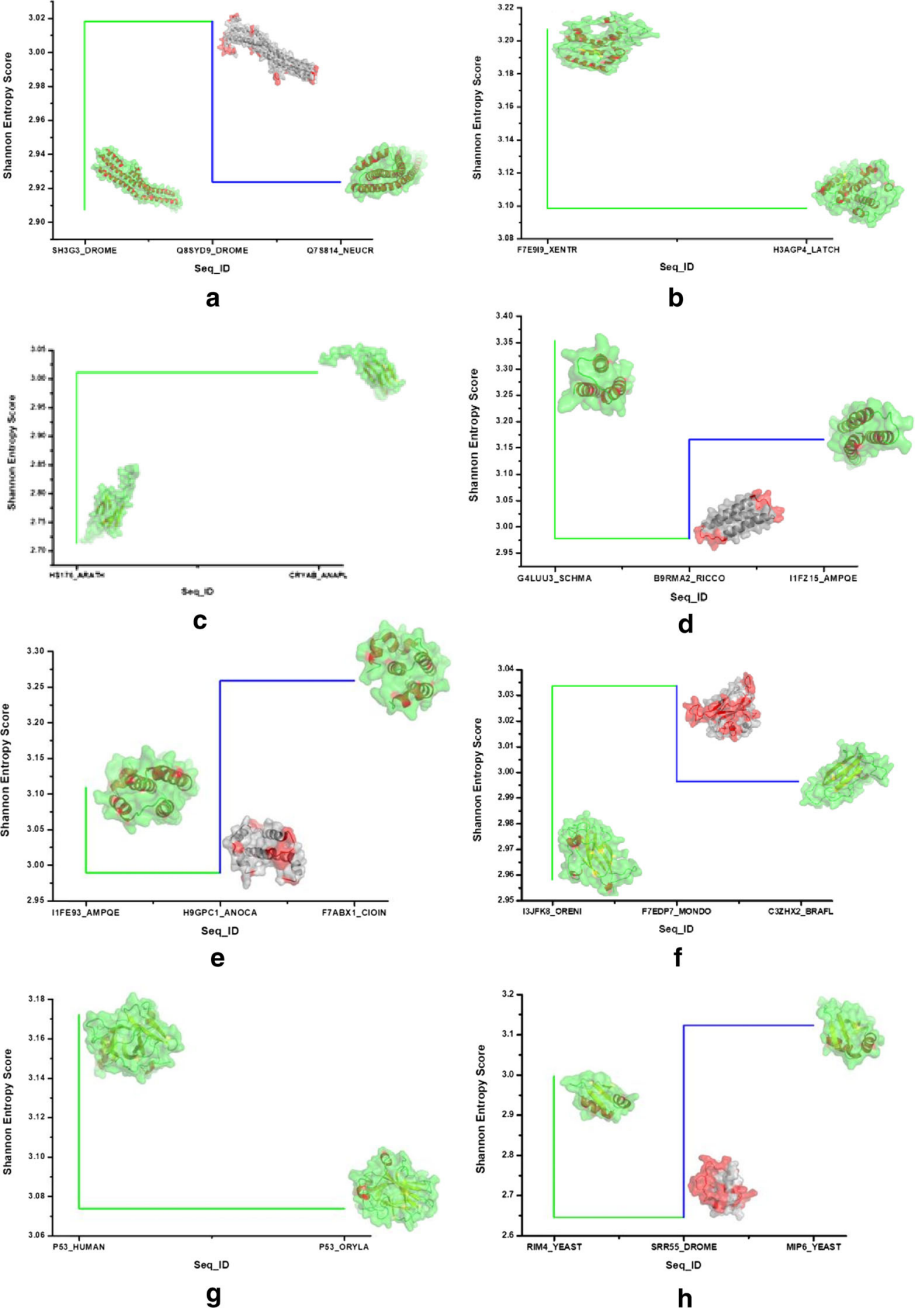
Representing the change in evolutionary trend in **a**. Endophilin-B1, **b**. Breast cancer type 2 susceptibility protein, **c**. Heat shock protein beta-1, **d**. BAG family molecular chaperone regulator 1, **e**. Apoptosis regulator Bcl-2, **f**. Calpain-type cysteine protease DEK1, **g**. Cellular tumor antigen p53 and **h**. RNA-binding protein 38 proteins reponsible for four cancer types such as breast cancer, prostate cancer, acute lymphoblastic leukemia and lymphoma respectively

We observed the transition points of transformation and considered those sequences for further analysis. In Fig. 2, a representative of each protein families was identified where the sudden deviation from the entropic score of consensus sequences occurred and reported their Shannon entropic changes along with the structures. Also, other two sequences of the protein families were mapped in their respective structures. From these structures, the deviation of structural changes along the entropic scores was easily visualized. Figure 2a and b represented the two proteins responsible for breast cancer. Similarly, Fig. 2c-h represented selected proteins responsible for prostate cancer, acute lymphoblastic leukemia and lymphoma respectively. The hydropathy index of those sequences was analyzed and validated with respect to entropic score.

**Fig. 3:**
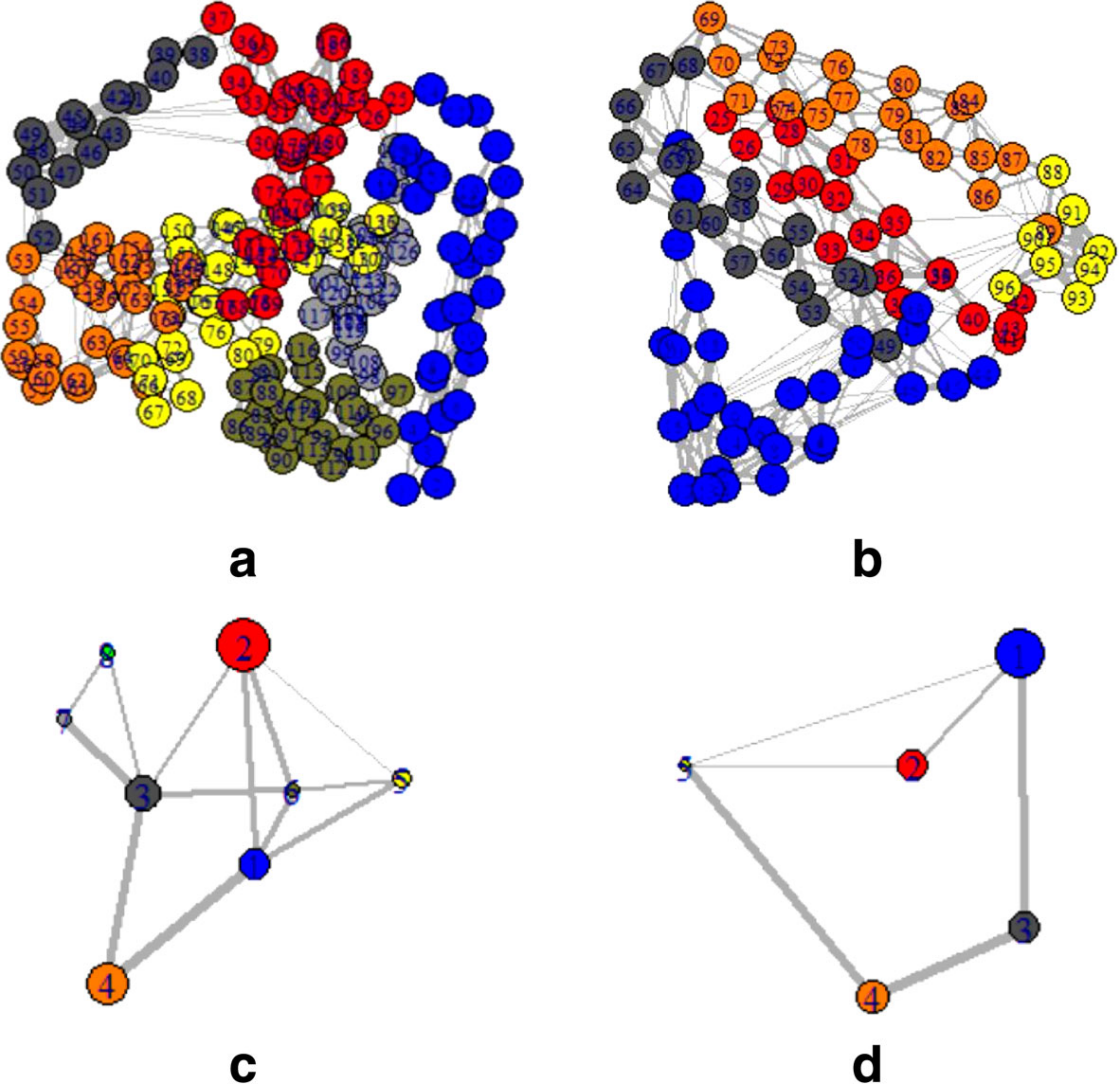
Structure network of Acute Lymphoblastic Leukemia for two spices such as **a**. Monodelphis domestica and **b**. Anolis carolinensis which represent high and low entropic score respectively. Similarly, the Community cluster network of **c**. Monodelphis domestica and **d**. Anolis carolinensis are shown

**Fig. 4:**
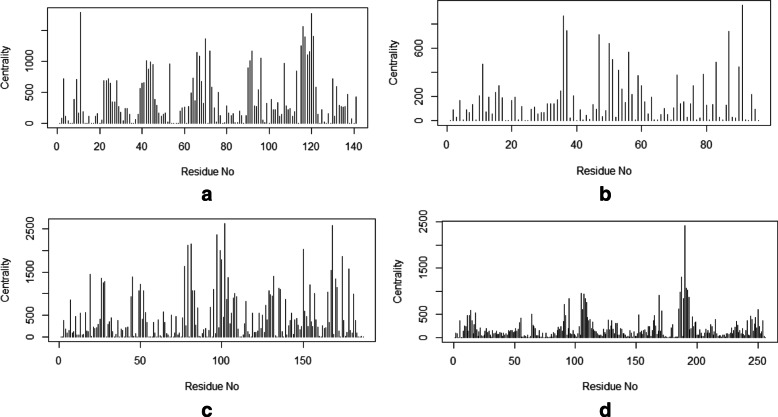
Betweenness centrality plot of Acute Lymphoblastic Leukemia for **a**. Monodelphis domestica and **b**. Anolis carolinensis which represent high and low entropic score respectively. Similarly, the plot is shown for two spices affected by breast cancer such as **c**. Xenopus tropicalis and **d**. Drosophila melanogaster

**Fig. 5:**
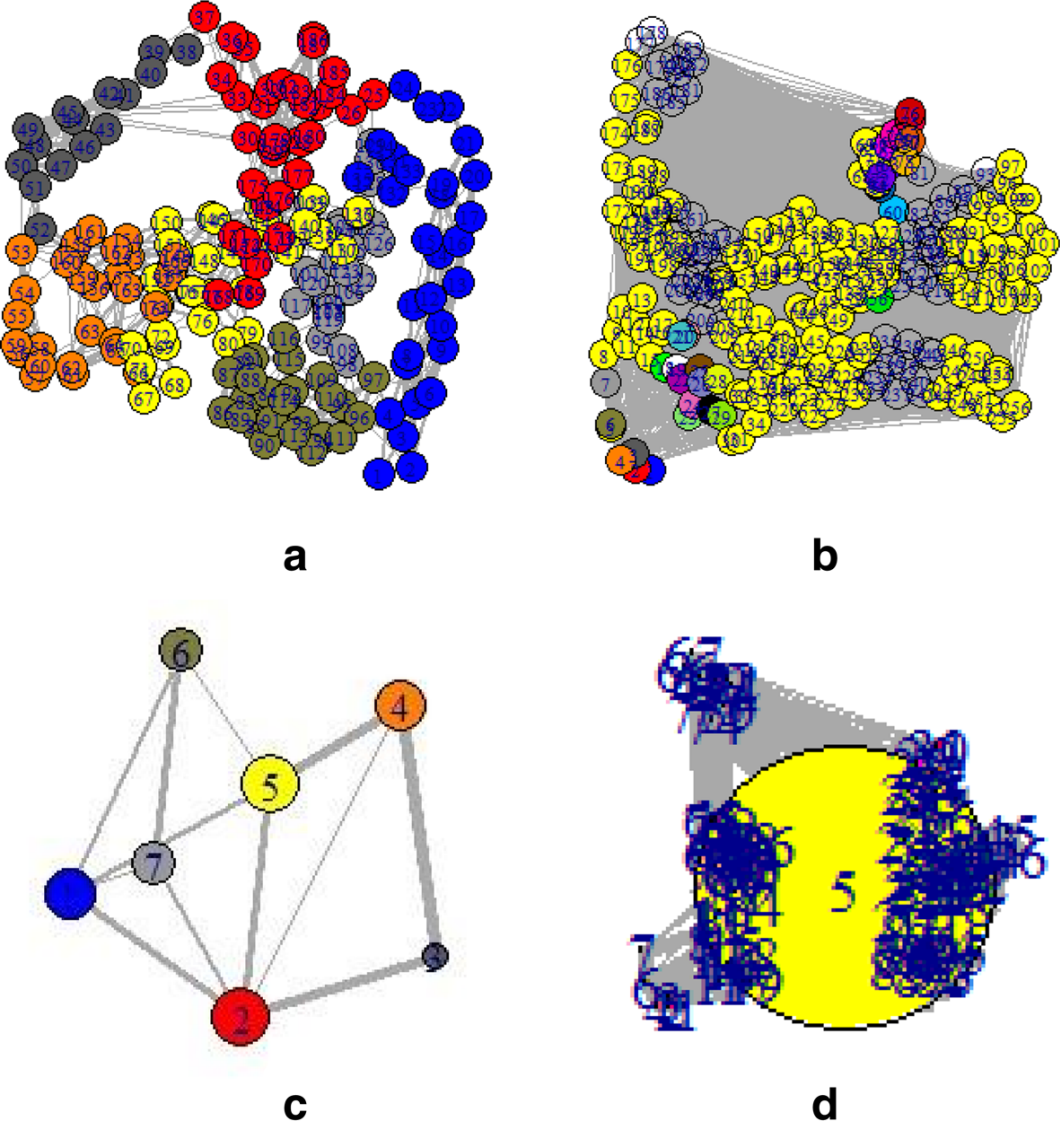
Structure network of Breast Cancer for two spices such as **a**. Xenopus tropicalis and **b**. Drosophila melanogaster which represent high and low entropic score respectively. Similarly, the Community cluster network of **c**. Xenopus tropicalis and **d**. Drosophila melanogaster are shown

**Fig. 6:**
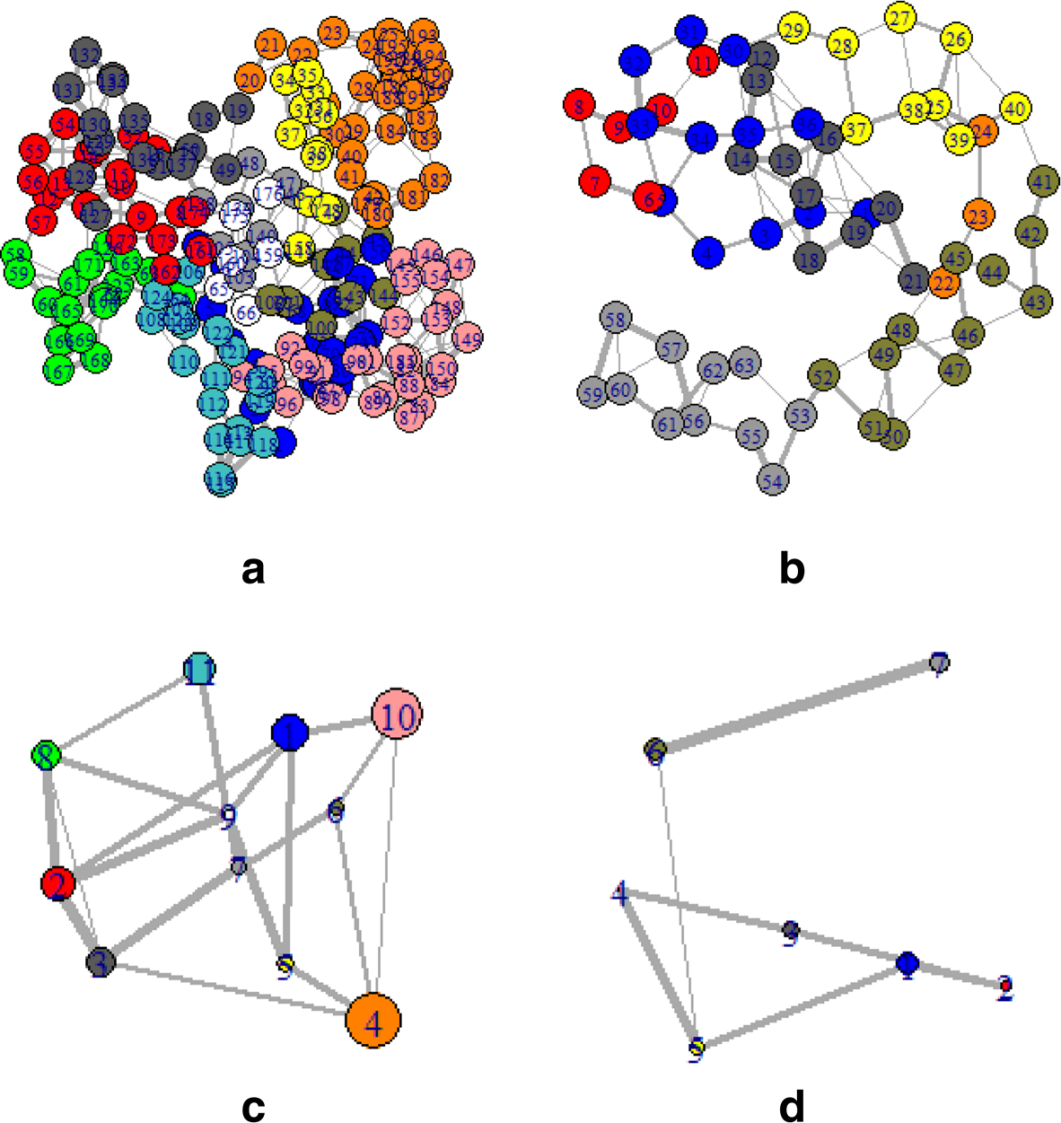
Structure network of Lymphoma for two spices such as Lymphoma for **a**. Homo sapiens and **b**. Drosophila melanogaste which represent high and low entropic score respectively. Similarly, the Community cluster network of **c**. Homo sapiens and **d**. Drosophila melanogaste are shown

**Fig. 7:**
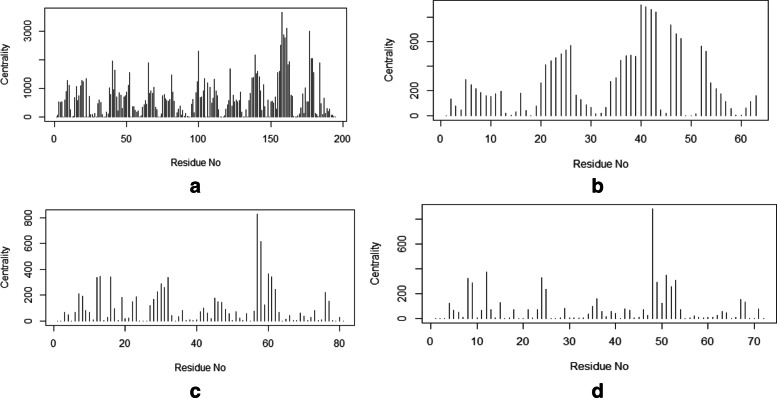
Structure network of Lymphoma for two spices such as Lymphoma for **a**. Homo sapiens and **b**. Drosophila melanogaste which represent high and low entropic score respectively. Similarly, the Community cluster network of **c**. Homo sapiens and **d**. Drosophila melanogaste are shown

**Fig. 8:**
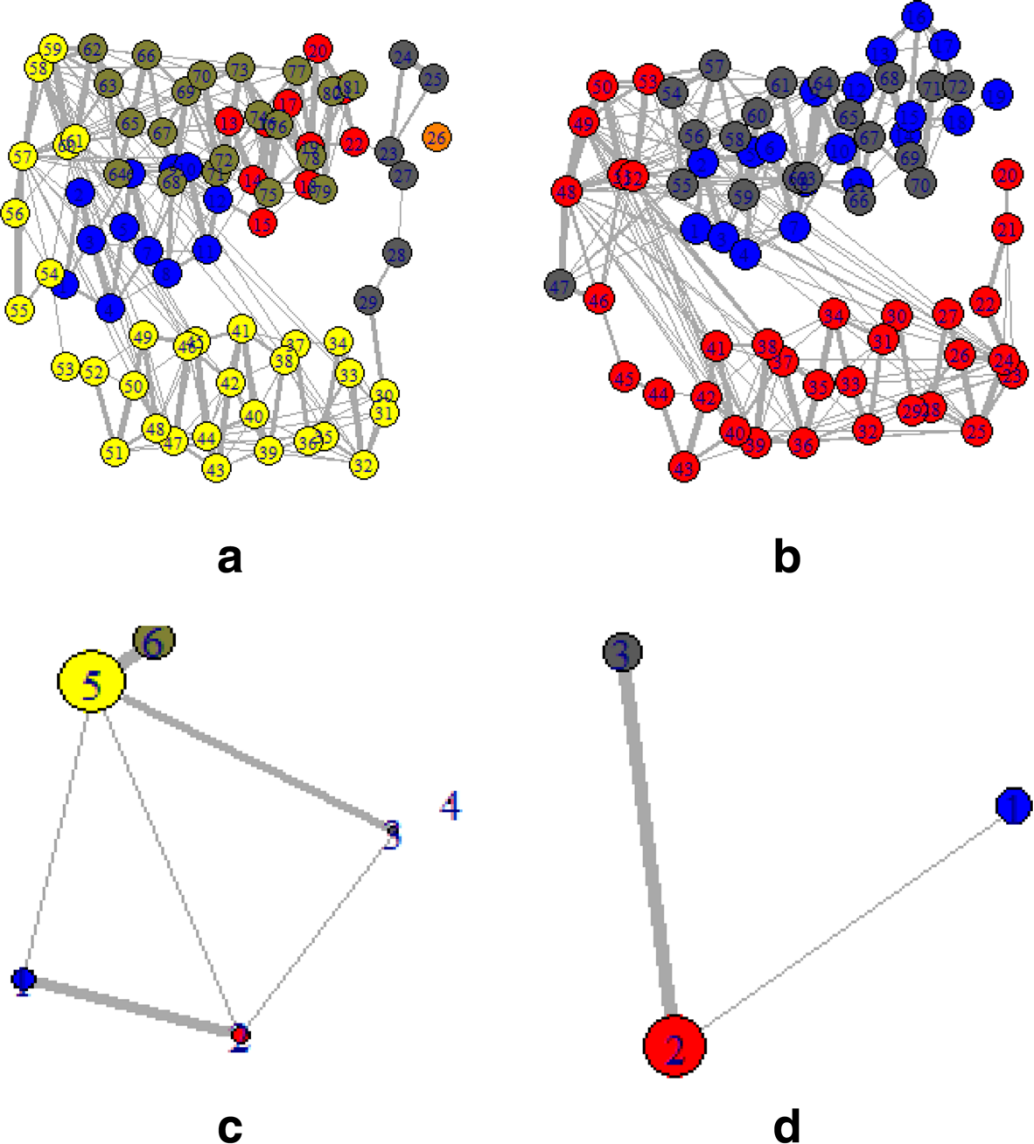
Structure network of prostate cancer for **a**. Aspergillus niger and **b**. Ricinus communis which represent high and low entropic score respectively. Similarly, the Community cluster network of **c**. Aspergillus niger and **d**. Ricinus communis are shown

To provide a comprehensive understanding of mentioned changes, the PDB structures of those particular sequences were also observed. Depending on the PDB, structure networks were shown along with the community cluster network and betweenness centrality plot. In Figs. 3-8, the structure network analysis, Community cluster network and betweenness centrality plots were shown for four diverse cancer types. Though we had performed the analysis of multiple proteins from multiple families, most diverse samples were shown in this article. From structure network analysis, the dependencies on residues at different secondary structura orchestrations could be observed. From the experimental outcomes, ordered structures had a diverse set of community clusters based on conservation of residue-residue interaction than disordered structure and also betweenness centrality graph was well distributed than disordered structures. Each directly coupled pair, obtained from structure space analysis, were found to be situated either in common cluster or in two densely connected clusters. The betweenness centrality was calculated to unveil the influence of a particular node on the internal dynamics of different structure.

## Discussion

In case of sequence biology, scoring of hydropathic index described the complexity of protein primary structure. It helped to understand the propensity of protein in terms of structural order or disorder. As mentioned before, each of the structurally affected human proteins and their family were chosen from four diverse cancer types. Observing the trend of Shannon entropic score of the consensus sequence of each family, structural transformation of the proteins could be considered as the reason behind diseased conditions. In Table 1, the Shannon entropic scores of all selected families were reported. Among them, only breast cancer and leukemia have one of each ordered family propensity. This fact justified the number of conserved structural motif of the family had ordered propensity. Hence most of the proteins of the family were ordered. Subsequently, the proteins with disordered propensities in consensus sequence scores were showing the disordered trend. Statistically, the entropic scores for consensus were significant. In Table 2, entropic scores of the human proteins were compared with their average hydrophobic index. Mostly proteins with disordered entropic scores were showing compatibility with average hydrophobic index. Hydrophobic index was justifying the spontaneous folding capacity of the protein. So lower hydrophobic index was indicating towards higher disordered propensity. Hence the compatibility between two different scoring systems could be clearly observed from Table 2.

**Table 2:** The hydrophobic index value of the selected proteins for four cancer types

Disease	Uniprot Id	Entropic score	Average score of
			hydrophobic index
Prostate cancer	P04792	2.96	0.60
Breast cancer	P38398	3.16	0.41
Acute lymphoblastic leukemia	Q8RVL1	2.94	0.58
Lymphoma	P04637	3.17	0.37
	Q9H0Z9	3.03	0.55

Thereafter, the sequence specific information was compared with the three-dimensional structures of proteins. In Fig. 3, two proteins of the leukemia were shown in terms of Gaussian network model based structure networks and their highly conserved community clusters. Similarly, in Figs. 5, 6 and 8, the structure network and community clusters were given for breast cancer, lymphoma and prostate cancer respectively. Depending on the number of shortest path on each residue, betweenness centrality plots were given in Figs. 4 and 7. From Fig. 4, the distribution of residual dependencies in terms of betweenness centrality for leukemia and breast cancer were given. The residual for the random leukemia protein sample in Fig. 4a, has justified the entropic score of its family. In Fig. 4a, the residual distribution was highly conserved at certain residual points whereas, in Fig. 4b, dependencies in terms of centrality scores were well distributed throughout the sequence. Though the family of Fig. 4b was maintaining ordered trend this particular protein structure was showing a disordered trend in terms of individual entropic score. That is why few higher peaks in the plot have been seen. Similarly, in Fig. 4c and d, the centrality plotting for breast cancer samples were given which were following the similar trend like leukemia samples. In Fig. 7, centrality distribution for lymphoma and prostate cancer were shown. Comparing with sequence information, the family with higher disordered propensity was showing conservation at certain residual points even in the ordered samples of the family. Hence the path of evolutionary transformations of the proteins from the family could be described from this observations. In human samples, the sudden structural changes were following the common mentioned path of transformations by disrupting the amount of average hydrophobic amino acids. Again the spontaneous folding capacity of the structure could be affected.

## Conclusion

In this article, we have proposed a method based on sequence complexity calculation of each protein families using Shannon entropic scoring for different malignancies. For four different cancer types viz., prostate cancer, lymphoma, acute lymphoblastic leukemia and breast cancer, eight different protein families were selected which structurally involved with the diseases. The objective was to observe the structural transformation of proteins in an evolutionary timespan. It was successfully shown that the entropic scoring based on amino acid distributions in the sequence helped to understand structured or unstructured propensity of proteins and their families. The results, obtained from entropic studies were complemented by hydrophobic indexing of the sequences. To map the sequence on structure, a structure space analysis was also performed. For each structure, the changes in residual dependencies were observed based on variation in betweenness centrality. Distribution of centrality for the structures were showing a compatible pattern with sequence dependent information. More precisely, structural orchestrations of proteins were varying with entropic scores accordingly. Finally, the experimental outcomes and comparative analyses suggested the evolutionary path of transformation in protein structures which could be comprehended by theoretical entropic scoring based on the conserved residual distribution in protein sequences.

## Additional files

Additional file 1 The HMM logo or signature of the selected protein families. (PDF 479 kb)

Additional file 2 Shannon entropy score of proteins under Endophilin-B1 family responsible for Breast cancer. (XLSX 10 kb)

Additional file 3 Shannon entropy score of proteins under Breast cancer type 2 susceptibility protein family responsible for Breast cancer. (XLSX 10 kb)

Additional file 4 Shannon entropy score of proteins under Heat shock protein beta-1 protein family responsible for Prostate cancer. (XLSX 10 kb)

Additional file 5 Shannon entropy score of proteins under BAG family molecular chaperone regulator 1 protein family responsible for Prostate cancer. (XLSX 16 kb)

Additional file 6 Shannon entropy score of proteins und er Apoptosis regulator Bcl-2 protein family responsible for acute lymphoblastic leukemia. (XLSX 15 kb)

Additional file 7 Shannon entropy score of proteins under Calpain-type cysteine protease DEK1 protein family responsible for acute lymphoblastic leukemia. (XLSX 15 kb)

Additional file 8 Shannon entropy score of proteins under Cellular tumor antigen p53 protein family responsible for lymphoma. (XLSX 9 kb)

Additional file 9 Shannon entropy score of proteins under RNA-binding protein 38 protein family responsible for lymphoma. (XLSX 11 kb)

## Abbreviations

DNA: Deoxyribonucleic acid
IDPs: Intrinsically disordered proteins
MSA: Multiple sequence alignment
NMA: Normal mode analysis
PDB: Protein data bank
SE: Shannon entropy
