# Supplementary material for: Understanding the evolutionary trend of intrinsically structural disorders in cancer relevant proteins as probed by Shannon entropy scoring and structure network analysis
Source: BMC Bioinformatics. 2019 Feb 4;19(Suppl 13):549. doi: 10.1186/s12859-018-2552-0 (PMC7394331; doi:10.1186/s12859-018-2552-0)
Supplement: Supplementary file 1 — The HMM logo or signature of the selected protein families. (PDF 479 kb) [file 12859_2018_2552_MOESM1_ESM.pdf]

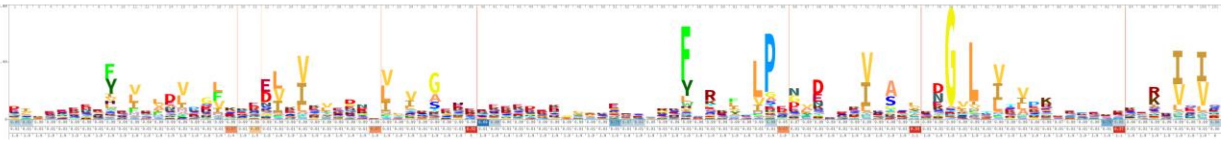

HMM Logo of Heat shock protein beta-1

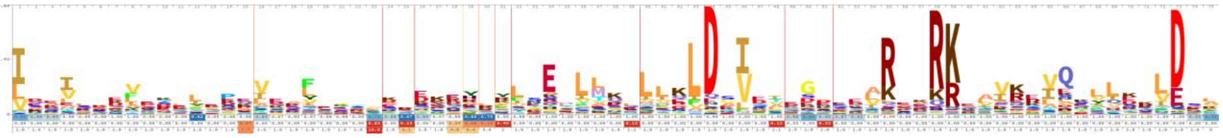

HMM Logo of BAG family molecular

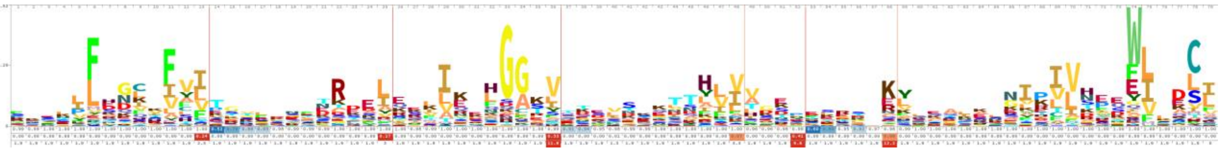

HMM Logo of Breast cancer type 2

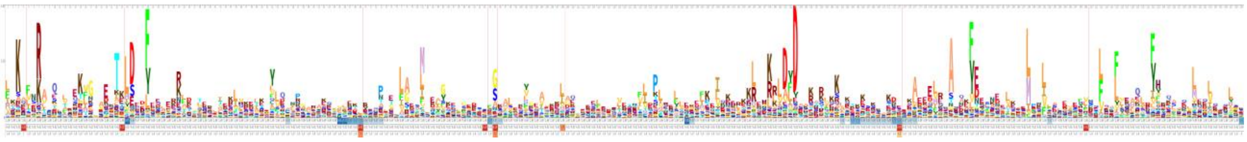

HMM Logo of Endophilin-B1

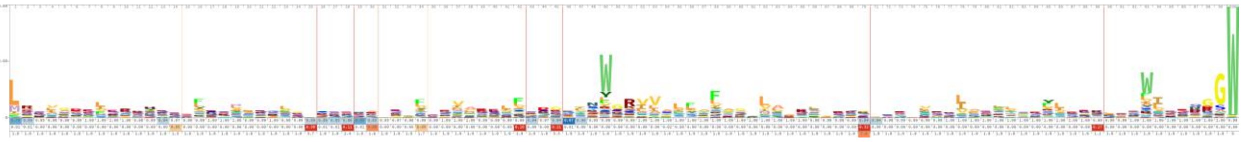

HMM Logo of Apoptosis regulator

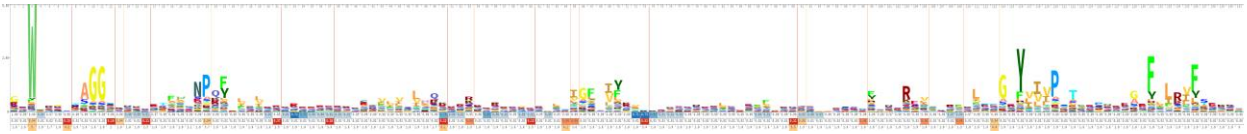

HMM Logo of Calpain-type cysteine

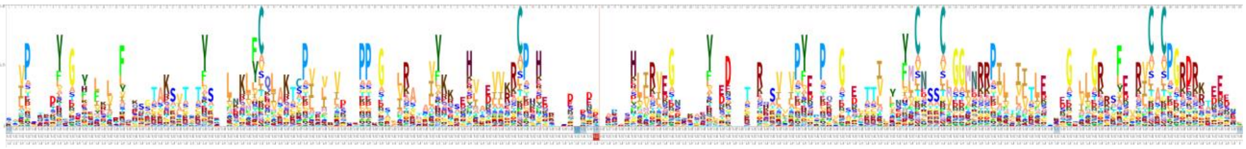

HMM Logo of Cellular tumor antigen p53

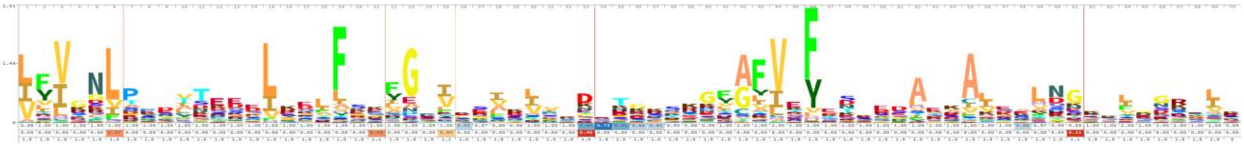

HMM Logo of RNA-binding protein 38
